# What side effects are problematic for patients prescribed antipsychotic medication? The Maudsley Side Effects (MSE) measure for antipsychotic medication

**DOI:** 10.1017/S0033291717000903

**Published:** 2017-04-19

**Authors:** T. Wykes, J. Evans, C. Paton, T. R. E. Barnes, D. Taylor, R. Bentall, B. Dalton, T. Ruffell, D. Rose, S. Vitoratou

**Affiliations:** 1 Psychology Department, Institute of Psychiatry, Psychology, and Neuroscience, King's College London, London, UK; 2 South London and Maudsley NHS Foundation Trust, London UK; 3 NIHR Biomedical Research Centre, South London and Maudsley NHS Foundation Trust and Institute of Psychiatry, Psychology, and Neuroscience, King's College London, London, UK; 4 Chief Pharmacist, Oxleas NHS Foundation Trust, Dartford, UK; 5 Centre for Psychiatry, Imperial College London, London, UK; 6 Pharmacy and Pathology, South London and Maudsley NHS Foundation Trust, London, UK; 7 Institute of Pharmaceutical Science, King's College London, London, UK; 8 Department of Psychological Sciences, University of Liverpool, UK; 9 Department for Psychological Medicine, Institute of Psychiatry, Psychology, and Neuroscience, King's College London, London, UK; 10 Health Service & Population Research, Centre for Implementation Science, Institute of Psychiatry, Psychology, and Neuroscience, King's College London, London, UK; 11 Psychometrics and Measurement Lab, Biostatistics and Health Informatics, Institute of Psychiatry, Psychology, and Neuroscience, King's College London, London, UK

**Keywords:** Medication side effects, self report, participatory methods, PROM, schizophrenia

## Abstract

**Background:**

Capturing service users’ perspectives can highlight additional and different concerns to those of clinicians, but there are no up to date, self-report psychometrically sound measures of side effects of antipsychotic medications.

**Aim:**

To develop a psychometrically sound measure to identify antipsychotic side effects important to service users, the Maudsley Side Effects (MSE) measure.

**Method:**

An initial item bank was subjected to a Delphi exercise (*n* = 9) with psychiatrists and pharmacists, followed by service user focus groups and expert panels (*n* = 15) to determine item relevance and language. Feasibility and comprehensive psychometric properties were established in two samples (N43 and N50). We investigated whether we could predict the three most important side effects for individuals from their frequency, severity and life impact.

**Results:**

MSE is a 53-item measure with good reliability and validity. Poorer mental and physical health, but not psychotic symptoms, was related to side-effect burden. Seventy-nine percent of items were chosen as one of the three most important effects. Severity, impact and distress only predicted ‘putting on weight’ which was more distressing, more severe and had more life impact in those for whom it was most important.

**Conclusions:**

MSE is a self-report questionnaire that identifies reliably the side-effect burden as experienced by patients. Identifying key side effects important to patients can act as a starting point for joint decision making on the type and the dose of medication.

## Introduction

Medication side effects, especially for antipsychotic medication, contribute to subjective ratings of mental health (Hayhurst *et al.* 2015) and can result in discontinuation or dose reduction by patients (Ashoorian *et al.* 2014; Morrison *et al.* 2015) which may produce multiple harmful effects. Side effects vary and include severe weight gain, impotence, insomnia, chronic sedation and a lack of concentration, all of which interfere with daily life activities. Close monitoring is important because they are associated with physical morbidity and mortality, poor adherence, and stigma, as well as a negative impact on quality of life. Joint decision-making on medication dose, or whether the benefits outweigh the risks or consequences, would be greatly enhanced if patients themselves were empowered to measure side effects outside a face-to-face contact (Morrison *et al.* 2016). Benefits include improved adherence and attitudes towards medication (Moncrieff *et al.* 2016) as well as prompt and appropriate intervention to minimise side-effect burden and prevent long-term physical health problems (Bauml *et al.* 2016). Over and above the health effects, such a measure could support a dialogue about specific medication type, dose or discontinuation and so contribute to a positive therapeutic relationship, which also has an effect on medication adherence (Sendt *et al.* 2015).

### Why do we need a self-report measure?

National audits of clinical records in UK mental health services have found deficiencies in the quality and frequency of recommended side-effect screening for patients prescribed antipsychotics in both community and inpatient settings (Paton *et al.* 2004; Taylor *et al.* 2004; Barnes *et al.* 2007). Observational studies (Turner, 1993; Jordan *et al.* 1999) and survey reports (Bennett *et al.* 1995; Gray *et al.* 2001) also found that, in clinical practice, mental health nurses tend to monitor only a limited number of antipsychotic side effects and to rely on general questioning and observation to detect medication-related problems. This data collection method is inaccurate as patients tend to underestimate adverse effects in response to such general questions (Yusufi *et al.* 2007; Cleary *et al.* 2012). The problems are no fewer in the clinical scientific evidence. Reviews of side-effect reporting conclude that most studies used no published side-effect ratings scales except for movement disorders (Pope *et al.* 2010) or only assessed a limited range of effects (Longden & Read, 2016). Spontaneous reporting of side effects important to patients is probably scarce given the likely misattribution of side effects to symptoms, forgetfulness or embarrassment about raising intimate effects.

### What should a measure include?

The comprehensive assessment of antipsychotic side effects presents several challenges, one being the diversity of potential problems, including anticholinergic and metabolic side effects and adverse effects on the motor, endocrine and cardiovascular systems. A recent review (van Strien *et al.* 2015) recommends self-report multi-domain scales to influence the clinical dialogue and allow sensitive questioning and clinical observation to produce a comprehensive assessment and to allow patients to report effects of importance to them which differ (Morrison *et al.* 2012) or be complementary to those of clinicians (Patel & David, 2007; Trujols *et al.* 2013).

### Why involve patients?

We will not rehearse in detail the notion that patient views are valid measures of treatment efficacy as there is plenty of evidence in the literature [e.g. Angermeyer *et al.* (2011)] including new data on side-effect reporting (Hayhurst *et al.* 2015). There is also evidence that service users regard patient-reported outcome measures (PROMs) as more appropriate and relevant than observer-rated measures (Crawford *et al.* 2011). PROMs are commonly used to measure the suitability of, and satisfaction with, treatment and therapies, including side-effect burden. However, patients have rarely been involved in their development. For instance, SMARTS, a pragmatic side-effect checklist (Haddad *et al.* 2014), is based on the views of psychiatrists and purports to be using lay language and yet no patient was involved in its design. The Glasgow Antipsychotic Side-effect Scale [GASS; Waddell & Taylor (2008)] was built only on information in the British National Formulary and decisions on medical importance made by the authors. This measure did include a patient focus group to agree acceptability, although no information is provided about whether the patients did find the questions acceptable. Given the evidence that patients have different priorities to clinicians, it is surprising that a True Patient Valued and Generated Reported Outcome Measure (PG-PROM) has not been developed. True PG-PROMs are those where patients are specifically involved in the development of the scale (including as researchers) as well as completing the measure by self-report. In addition to the obvious acceptability benefits, they are also likely to cover a wider list of patient valued side effects that may drive adherence and treatment engagement. A side-effect scale that includes such items will allow clinicians access to previously unrated side effects that may predict treatment response.

The participatory methodology for the development of True PG-PROMs is now well described [e.g. Rose *et al.* (2011)]. Development includes psychometric assessment especially given their current weaknesses (Ashoorian *et al.* 2014; van Strien *et al.* 2015). For instance, Wolters *et al.* (2009) report weak psychometric properties for the Drug Attitude Inventory [DAI; Hogan *et al.* (1983)]. The Liverpool University Neuroleptic Side Effect Rating Scale [LUNSERS; Day *et al.* (1995) potentially overestimates antipsychotic side-effect frequency (Wolters *et al.* 2009) and although it did involve patients in its development and has good psychometric properties (van Strien *et al.* 2015) is now out dated. In addition to moderate psychometric properties (van Strien *et al.* 2015) most studies had small samples affecting confidence in the supporting data. For instance, the GASS (Waddell & Taylor, 2008) test-retest measure is based only on 17 respondents.

For personalised medicine, a psychometrically sound measure would allow concurrent side effects from large populations to aid the identification of biomarkers of treatment effects quickly. Our aim is to develop such a sound PROM of antipsychotic medication side effects using innovative participatory methodology (Rose *et al.* 2011) to capture the patient perspective and more accurately reflect their priorities (Faulkner & Thomas, 2002; Trivedi & Wykes, 2002) which are likely to be different to those of clinicians. Identifying patient-valued negative effects might then drive the development of new treatments as well as the prescription of those medications already proven to be efficacious.

## Method

### Design

The study employed mixed methods with qualitative approaches for measure generation and quantitative methods to evaluate the psychometric properties. The Maudsley Side Effects (MSE) measure were developed in three phases: (1) generating the measure, (2) assessing reliability and other properties and (3) evaluating validity.

### Participants

Seven psychiatrists with a psychopharmacology background and two pharmacists from the UK, USA and Spain with a known expertise in side effects took part in the Delphi exercise.

For all other parts of measure development, we recruited participants with a clinical diagnosis of schizophrenia (according to case records), who were in touch with mental health services, aged 18–65 years and had taken antipsychotic, but not antidepressant, medication for a minimum of a month. We recruited participants from outpatient clinics, inpatient units and clozapine clinics in three waves:

• ***sample 1*** (N15) took part in the focus groups and the expert panel;
• ***sample 2*** (N43) completed the draft MSE on two occasions, feasibility questionnaire and detailed measures of clinical state;
• ***sample 3*** (N50) completed the MSE and another patient reported side-effect measure.

Ethical approval was given by the London Dulwich Ethics Committee (12/LO/2034).

### Measures

(1) Demographic and clinical data;
(2) Clinical state: (i) *Brief Psychiatric Rating Scale* (Ventura *et al.* 1993), an interview measure identifying mainly psychotic symptoms, and (ii) the General Health Questionnaire [GHQ-12; Goldberg (1992)] and the Short Form Health Survey Version 2 [SF-36v2; Ware *et al.* (2000)], self-report measures of general health with no questions relating to psychosis;
(3) GASS (Waddell & Taylor, 2008) a self-report measure of antipsychotic side effects;
(4) Feasibility questionnaire used in other measure developments [e.g. Evans *et al.* (2012)], which assesses accessibility, acceptability and interest.

### Procedure

#### Phase 1: Generating the draft measure

(i) ***Delphi consultation***: An initial bank of items was drawn from three existing self-report side-effect measures: The Antipsychotic Non-Neurological Side-Effects Rating Scale [ANNSERS: (Yusufi *et al.* 2005; Ohlsen *et al.* 2008)], GASS (Waddell & Taylor, 2008) and LUNSERS (Day *et al.* 1995). We undertook a Delphi exercise to determine the relevance and importance of each item based on up to date knowledge of current antipsychotic medications. We fed back initial item ratings and group means to each participant who re-rated them. Following this consultation, we recalculated the mean responses and used them to determine important items to include.
(ii) ***Service user consultation***: Two focus groups met to discuss their experiences of antipsychotic medication side effects and to comment on the items drawn from the Delphi exercise. Two service user researchers, one with extensive experience of using antipsychotic medication, ran the focus groups. Each group was audiotaped, transcribed and then analysed using nVIVO10. The comments and themes generated a draft measure and a service user expert panel commented on the content, language and format.

### Phase 2: Feasibility and item inclusion

Our expert panel of service users and pharmacologists considered low frequency items from sample 2 measure completion for future retention. Feasibility questionnaire data determined acceptability.

### Phase 3: Psychometric analysis

The measure will be used to generate discussion on patient valued items but four total scores can be extracted to summarise the information:

(a) Total side effects endorsed (range: 0–53).
(b) Total intensity by summing the 4-point Likert life intensity items (refers to the selected side effects, with range: 0–159).
(c) Total distress by summing the binary distress items (refers to the selected side effects, with range: 0–53).
(d) Total life-impact by summing the 4-point Likert life impact items (refers to the selected side effects, with range: 0–159).

We conducted psychometric properties analyses at both the item and subscale level. For the total scores, parametric methods (Pearson's correlation coefficient r and *t* test for the equality of means) and their non-parametric analogues were used (Spearman's rho and Wilcoxon test, respectively) depending on whether the scores were normally distributed.

We investigated different forms of both reliability and validity. For *reliability*, internal consistency was evaluated using Cronbach's alpha (Cronbach, 1951). For stability (*test–retest reliability*) sample 2 participants completed the new measure twice within an interval of 6–8 days. We used Cohen's kappa [κ; (Cohen, 1960)] to assess item level agreement. For ordinal items, weighted kappa [κ_w_; (Cohen, 1968)] is reported and we have used the Landis and Koch (Landis & Koch, 1977) interpretations (negative values no agreement, >0.8 almost perfect agreement). We used correlation coefficients between the two time points and the differences between the corresponding means when evaluating the total scores.

We evaluated *concurrent, content validity* in both samples 2 and 3. We assessed *convergent* and *divergent* validity in sample 2 via the association of total MSE scores with BPRS, GHQ and SF-36v2. We used the GASS scale in sample 3 to estimate the convergent validity and for *criterion validity*, we evaluated the presence of a likely side effect (drooling) with the prescription of clozapine in both samples. Finally, the inter-correlations between the summary scores and the effect of age and gender provided further evidence towards the content validity of the MSE.

All analyses were conducted using SPSS 23 along with irr (Gamer *et al.* 2010) and psych (Revelle, 2010) packages in R 3.0.2.

## Results

### Participants

All samples had similar demographic characteristics. (see Table 1). The median BPRS score of sample 2 was 45, which is close to the median score of 47.3 noted in individuals with established schizophrenia (McCleery *et al.* 2014). Clinical state did not change significantly across the two time points (Web-Table S1; *r* = 0.85 *p* < 0.001; median scores: T1 45, T2 48 and 46% with 0 change).

**Table 1.** Demographic and clinical characteristics

	Phase 1 (sample 1)		Phases 2 and 3			
	Focus group and expert panel		Sample 2 (*N* = 43)		Sample 3 (*N* = 50)	
	*N*	%	*N*	%	*N*	%
Gender						
Men	3	20	27	63	28	56
Women	12	80	16	37	22	44
Ethnicity						
White	12	80	14	32	18	36
BME	3	20	29	68	32	64
	Range	Mean (s.d.)	Range	Mean (s.d.)	Range	Mean (s.d.)
Age (years)	29–56	41.2 (8.7)	24–64	45.8 (9.9)	28–64	45.7 (8.9)
BPRS			26–73	46.0 (12.9)		
GHQ			0–23	11.1 (5.4)		
SF36-physical			21–64	44.7 (10.1)		
SF36-mental			25–65	44.0 (10.2)		

### Phase 1: Measure generation

The Delphi exercise identified items of: (i) uncertain validity, (ii) detected only by observation or (iii) duplication and these items were removed. Other items were clarified and additional ones related to newer antipsychotic medications added. Following these changes, the research team (including the service user researchers) reworded all questions into a simple, easy-to-read format.

Prompted by the list of side-effects and following a discussion of personal experiences, focus group participants removed a duplicate item, generated eleven new items and advised on presentation and scaling. The expert panel (*n* = 6) added one new item and a comments box and considered the questionnaire both comprehensive and the right length.

At this point, there were 54 items each with three ratings: intensity (0-not at all, 1-mild, 2-moderate, 3-severe), distress (yes or no) and life impact (4-point Likert scale). Two additional items noted the most important three side effects and the balance of benefits of taking medication.

### Phase 2: Feasibility and draft items

In sample 2, the side effects reported most often (see Web-Table S2) were ‘feel tired’ (77%), ‘put weight on’ (70%), ‘passing urine’ (67%), ‘thirsty’ (67%) and ‘memory issues’ (65%) and very similar results were obtained in the replication group (sample 3 Web-Table S2). One low-frequency item was dropped as it had less value and was difficult to self-report leaving a 53-item scale to assess psychometric properties. MSE has a Flesch reading ease score of 103 where scores of 90–100 are regarded as ‘very easy’ to understand (Flesch, 1948). MSE was acceptable to the vast majority of the sample (99% found it easy to understand, 93% easy to complete and 93% an appropriate length). Three service users disliked completing the measure and 20% found some questions distressing, which were those associated with sensitive intimate issues – sexual problems, enuresis and constipation. It takes about 15 min to complete.

### Psychometric analyses

#### Descriptive data

The mean total number of side effects reported was 21 out of 53. Intensity, life impact and distress scores were skewed towards the lower end of the scale (see Web-Table S3). As expected a higher side-effect burden correlated with more distress and life impact and all these correlations were statistically significant (see Web-Table S4). As anticipated, neither age nor gender (Web-Table S5) affected side-effect endorsement (*p* > 0.1 in all cases) and all these results were replicated in sample 3 (Web-Tables S6 and S7).

#### Reliability

Cronbach's alpha for the total side-effects score was 0.96, indicating very high correlations between items. At the item *level*, the weighted Kappa coefficient indicated at least fair agreement for intensity items. Non-significant coefficients emerged only for ‘fits’, ‘rash’ and ‘catatonia’ side effects, probably due to their low frequency (see Web Table S6). At the *subscale level*, all scores were highly correlated (0.81–0.96) between the two time points and there were no statistically significant differences in the mean scores, indicating the instrument's good test–retest reliability (see Web Table S2). Symptoms did not affect reliability as it was stable for both high and low BPRS scorers [high scorers (above the median – 45): *ρ* = 0.85, *p* < 0.001; low scorers (below median): *ρ* = 0.94, *p* < 0.001].

#### Validity assessment

(i) We established *concurrent convergent validity* by comparing the side-effects summary scores with clinical state measures (sample 2). As expected, a greater number of side effects were related to poorer measures of general mental health (GHQ, SF-36 mental component) and physical health (SF-36) as items overlap (see Table 2).
  But our specific psychosis mental state measure did not have overlapping items so should not be related. We investigated side-effect reporting in the low and high BPRS scorers and found evidence of *discriminant validity* as the two BPRS groups did not differ in side-effect reporting (total side effects mean 22.7 *v.* 19.3; *Z* = 0.86, *p* = 0.39; intensity median 43.1 *v.* 29.7 *Z* = −1.26, *p* = 0.21; distress median 29 *v*. 19; *Z* = −0.95, *p* = 0.35). However, high BPRS scorers, as expected, reported significantly higher life impact (median 9 *v*. 2; *Z* = −2.858, *p* = 0.004) given that higher symptom levels plus the side effects are likely to have the highest impact on everyday life.
(ii) We established *concurrent convergent validity* between GASS and MSE (sample 3; Table 3). At the similar *item level*, the agreement in endorsement varied from 70% to 90%. For *distress* agreement ranged from 73% to 100%. All weighted kappa coefficients were significant and indicated fair to substantial agreement. At the *total score level* (all items included in both scales), the MSE and GASS subscales were highly correlated (total side effects: *r* = 0.8, intensity: *r* = 0.8 and distress: *r* = 0.7, *p* < 0.001 in all cases).

**Table 2.** Correlation coefficients between the MSE scores and SF-36 and GHQ scores

	SF-36 Physical		SF-36 Mental		GHQ	
	*r*	*p* value	*r*	*p* value	*r*	*p* value
Total effects	−0.4	0.003	−0.5	0.001	0.5	<0.001
Intensity	−0.5	<0.001	−0.5	<0.001	0.5	0.001
Life Impact	−0.7	<0.001	−0.4	0.009	0.5	0.004
Distress	−0.4	0.008	−0.4	0.019	0.2	0.219

**Table 3.** Similar Item level agreement assessed for items with at least 15 individuals reporting on the MSE

Side effect (item number GASS-MSE)	Intensity^a^		Existence				Distress		
	*N*	Weighted kappa (*p*)	*N* present GASS (%)	*N* present MSE (%)	%	kappa (*p*)	*N*	%	kappa (*p*)
Felt zombie (2–40)	50	0.6 (<0.001)	14 (28)	16 (32)	88	0.7 (<0.001)	12	100	1.0 (0.001)
Dizzy (3–20)	50	0.6 (0.003)	25 (50)	20 (40)	74	0.5 (0.001)	16	75	0.5 (0.049)
Heartbeat (4–14)	50	0.5 (<0.001)	20 (40)	20 (40)	76	0.5 (<0.001)	14	79	0 (0.672)
Shaky (6–15)	49	0.6 (<0.001)	15 (30)	15 (30)	84	0.6 (<0.001)	11	100	1.0 (0.001)
Restless (7–41)	49	0.8 (<0.001)	21 (42)	20 (40)	90	0.8 (<0.001)	18	94	0.9 (<0.001)
Drooling (8–19)	50	0.7 (<0.001)	29 (58)	31 (62)	84	0.7 (<0.001)	26	85	0.6 (0.001)
Slow move (9–24)	50	0.5 (0.001)	22 (44)	27 (54)	70	0.4 (0.003)	17	82	0.6 (0.005)
Blurry vision (11–21)	47	0.8 (<0.001)	21 (42)	20 (40)	89	0.8 (<0.001)	18	78	0.5 (0.034)
Dry mouth (12–5)	48	0.6 (0.001)	21 (42)	22 (44)	81	0.6 (<0.001)	17	77	0.2 (0.432)
Feel sick (14–42)	50	0.6 (<0.001)	16 (32)	16 (32)	84	0.6 (<0.001)	12	83	0.6 (<0.001)
Weight (22–23)	39	0.4 (<0.001)	24 (48)	23 (46)	92	0.8 (<0.001)	22	73	0.4 (0.070)

a Considered if the item was endorsed on both scales.

For *concurrent criterion validity*, we measured the effects of a side effect most often associated with Clozapine prescription, drooling. These results were replicated in sample 3 (N50), and those prescribed Clozapine (N30; 60%) were more likely to report drooling and when they experienced drooling it was more intense compared with those prescribed other medications [(i) frequency of reporting 80% *v*. 35%; χ^2^ = 10.314, df = 1, *p* = 0.001; higher intensity when reported, median: 1.6 *v*. 0.7; Mann–Whitney *U* = 157.5, *p* = 0.003]. The pattern of results was the same for sample 2 although there were fewer people prescribed clozapine (N9; 21%). We tested for differences in distress and life impact after merging the two samples but no statistically significant differences emerged (χ^2^ = 0.570, df = 1, *p* = 0.450) or life impact (median: 1 in both cases; Mann–Whitney *U* = 183.0, *p* = 0.130) suggesting that drooling is distressing and affects life, whichever drug produced the effects.

### The most important side effects

The pattern of most important side effects was the same samples 2 and 3 with only one difference, drooling was chosen more often in sample 3 selecting this item (21% *v*. 11.6%; χ^2^ = 5.488, df = 1, *p* = 0.019) probably because it contained more people prescribed clozapine.

Participants (merged sample N93) endorsed 42 of our 53 items as one of the three most important side effects (see Web Table S8) providing further validity for the breadth of scale items. The three most often mentioned were feeling tired, drooling and putting on weight selected by 16–23% of the total sample. The remaining items were selected by <9% suggesting that what bothers an individual is idiosyncratic.

#### What determines an individual's choice of the most important side effect?

A participant's choice did not follow the endorsement frequency. For instance, 62% identified memory problems, but they were only mentioned as ‘most important’ by 16%. As many ‘most important’ items were low frequency, we concentrated on the side effects most frequently reported – feeling tired, drooling and putting on weight. Choosing tiredness or drooling was not related to how an individual rated its severity, distress or life impact. However, for ‘putting on weight’ those who mentioned it as most important also rated it as more severe (60% *v*. 29.4%, χ^2^ = 6.384, df = 1, *p* = 0.041), having more life impact (70% *v*. 20.4%, χ^2^ = 12.432, df = 1, *p* = 0.006) and being more distressing (90% *v*. 27.5%, χ^2^ = 13.926, df = 1, *p* < 0.001).

## Discussion

We derived the new measure from the published literature on antipsychotic side-effect rating scales, with the participation of experts from psychiatry and pharmacy, service users (including service user researchers) and methodologists. We adopted this process in order to ensure that we captured the sorts of side effects that service users’ value. In addition, we also had the advantage of updated knowledge on the side effects of newer antipsychotic medications. Our final measure consists of 53 side-effect items, each with three associated questions regarding intensity, distress and life impact. The measure covers a range of antipsychotic side effects, including metabolic, sexual and anticholinergic effects. Items are in the words used by patients so that they are easily understood. The final MSE is a measure of an individual's side-effect burden, not a checklist of the drug-related phenomena present.

The measure was acceptable and feasible, and demonstrated strong psychometric qualities for both reliability and various forms of validity in its self-report form. Importantly, the severity of psychotic symptoms did not affect side-effect endorsement suggesting it is appropriate for those who are more acutely unwell. This is the first time a measure, acceptable to service users and with high-quality psychometric data have been developed and it therefore overcomes the problems of previous measures. The MSE follows the recommendations of recent reviews in being more comprehensive (multi-domain) (van Strien *et al.* 2015) with strong psychometric and appropriate measures (Wolters *et al.* 2009; Stomski *et al.* 2016). Some side effects may look different to those described in other measures (e.g. feel like a zombie), but this is because the items were generated by patients themselves.

### How does our measure differ from others?

Apart from updating the content (a constant process) we adopted a participatory approach with input from clinicians and, importantly for a self-report measure, service users. We are not the first to involve service users in the development of a side-effect measure as the authors of the GASS (Waddell & Taylor, 2008) also consulted them but only in one focus group to rank the item acceptability. Our involvement was more extensive, and produced noticeable changes, including adding in side effects that were valued by them and considered to be qualitatively different, e.g. items relating to sleep or tiredness were divided into feeling tired, sleeping too much, difficulty staying awake, hard to fall asleep and hard to get out of bed. It is notable that GASS is the only measure to include distress (as we did) but our measure is the first to include life impact. Many measures (e.g. LUNSERS; Day *et al.* 1995) also use red herring items but in the initial review by our psychiatry and pharmacy experts many of these red herrings were either subsequently indicated as an actual side effect or were so transparent that they were likely to irritate service users. Hence, we excluded them from the beginning.

The issue of length as well as breadth produces different responses from clinicians and patients. The view from clinicians is that a short questionnaire is preferable so most clinician generated scales are often <20 items. In contrast, scales involving patients, e.g. LUNSERS and SRA (Day *et al.* 1995; Wolters *et al.* 2006) are the longest but even so LUNSERS was picked as a favoured outcome by patients (Crawford *et al.* 2011). A balance needs to be struck, but multi-domain scales are likely to be longer if they are to be comprehensive. The fact that so many different items were mentioned in the three ‘most important’ side effects suggests that it would be hard to reduce the scale. Despite being twice as long as another comparable measure MSE only takes about 15–20 min to complete and importantly is acceptable to service users.

### Which side effects are important to patients?

The choice of the ‘most important’ side effects seems to be idiosyncratic and it would be difficult to determine them from their frequency. Severity, distress nor life impact predicted the endorsement of two of the top three most important items, tiredness and drooling. However, ‘putting on weight’ was related to distress and life impact, replicating a recent survey (Ashoorian *et al.* 2015) where it was the most ‘bothersome’ side effect.

Clinicians are obviously concerned about side effects that have health consequences, but these are often only obvious on physical examination, with blood tests and detailed targeted questions. No self-report scale can be a substitute for such clinical examination. For clinical use, the opportunity for service users to describe the most important side effects, as well as listing those present will enable a more informed discussion on reducing patient-valued unwanted effects. These may be effects with a large life impact, but do not necessarily have medical consequences, at least in the short term, and therefore in a clinical encounter they could be overlooked. Changes in the perceived value of side effects over time may reflect changes, not in medication dose or tolerability, but in the process of recovery. For instance, if you are staying at home a slight shakiness might not be as important to a service user, but the importance of this side effect will change if the service user then needs to use a smartphone or computer for work. These changes need to be captured, as MSE does, as they highlight areas for clinical discussion about the risks and benefits of medications, which can improve the therapeutic alliance. These discussions are also vital because recent studies suggest that poor adherence in the short term may be only loosely associated with longer lasting negative attitudes which are influenced by side-effect burden (Hui *et al.* 2016).

Patient-value for negative effects is also important for treatment development. Currently, patient perspectives are used only at the end of the development, but perhaps drug developers can use evidence of side-effect value in considering which of several compounds to take into phase 1 or 2. The result of such developments might be a shift in patient involvement not to the dissemination and adoption sections of the translational pipeline but much closer to the bench so research is driven also by the patient perspective (Callard *et al.* 2012).

We hope that MSE will enhance clinical engagement, an important issue for treatment adherence and recovery. MSE can also provide information over time on the effects of dose changes on patient rated important items and will also allow tracking of side-effect burden so providing clinical utility too.

### Strengths and limitations

A study strength is our service user input and our extensive testing of psychometric properties to demonstrate good reliability and validity. This measure will not substitute for clinical observation, as it is limited to side effects that are easily self-reported. Medically important effects will require clinical observation. Although we found no relationship between symptoms of the disorder and side-effect endorsement, some side effects endorsed might not be related to medication but may be related to unpleasant effects of the disorder, e.g. agitation rather than drug-induced akathisia. Alternatively, a side effect may not be drug related, but may be related to an underlying physical cause requiring further investigation of the aetiology. We therefore emphasise that this measure is one tool, but not the only tool, to inform decisions about medication; it is an adjunct to, but not a replacement for, joint decision-making. We included a group of service users who were middle aged and drawn mainly from outpatient services and so future studies need to investigate both older and younger groups and inpatients who may have slightly different views on the importance of some side effects.

### Clinical uses

We consider this measure a valuable tool to support clinicians in their discussions and negotiations about medications. These discussions may be aimed at changing the type or dose of medication, or to discuss potential discontinuation. Sometimes side effects are not reported in a clinical encounter because they are considered unavoidable or for which there are no obvious solutions. They may not also be reported because the service user is embarrassed to discuss them. MSE should facilitate such encounters and allow both clinicians and service users to monitor the effects of dose changes or changes of drug on side effects over time. We suggest that this measure ought to be completed at least once per year and more frequently to monitor specific changes in medication. We will be including this measure on a patient portal – myhealthlocker™ – so data on side effects can also feed into information on the efficacy of medications across groups of patients and within particular services.

## Conclusion

We have produced a self-report side-effect burden measure for antipsychotic medication, which allows patients to reflect on the effects most important to them. It is feasible, acceptable and has good psychometric properties. This is the sort of measure voted as preferred by patients (Crawford *et al.* 2011), and which has the chance to support clinical discussions, improve therapeutic engagement, in addition to allowing clinicians an insight into their patients’ changing perspectives on side effects that have a large life impact.

## Supplementary material

For supplementary material accompanying this paper visit http://dx.doi.org/10.1017/S0033291717000903. click here to view supplementary material
