# Supplementary material for: What side effects are problematic for patients prescribed antipsychotic medication? The Maudsley Side Effects (MSE) measure for antipsychotic medication
Source: Psychol Med. 2017 Apr 19;47(13):2369–78. doi: 10.1017/S0033291717000903 (PMC5820531; doi:10.1017/S0033291717000903)
Supplement: Supplementary file 1 [file S0033291717000903sup001.docx]

Web Table 1: Test retest correlation coefficients and mean differences for GHQ, BDI, BPRS and SF36 total scores.

|  | **Correlation**  **between**  **time points** | | **Mean difference**  **between time points (t1-t2)** | | |
| --- | --- | --- | --- | --- | --- |
|  | **r** | **p-value** | **Mean difference (S.E.)** | **Test statistic** | **p-value** |
| ***BPRS*** | 0.85 | <0.001 | -2.0 (7.5) | t=-1.722, df=40 | 0.093 |
| ***GHQ*** | 0.66 | <0.001 | 0.1 (4.6) | t=0.099, df=42 | 0.921 |
| **SF36-physical** | 0.85 | <0.001 | -1.0 (5.8) | t=-1.119, df=40 | 0.270 |
| **SF36-mental** | 0.71 | <0.001 | -0.9 (7.8) | t=-0.738, df=40 | 0.465 |

Web Table 2: Number of participants that endorsed each side-effect, by sample

| **Item** | **Side effect** | **Sample** | | | |
| --- | --- | --- | --- | --- | --- |
|  |  | ***Sample 2 (Ν=43)*** | | ***Sample 3 (Ν=50)*** | |
|  |  | **N** | **%** | **N** | **%** |
| Q01 | Headaches | 22 | 51 | 22 | 44 |
| Q02 | Not feeling emotions | 20 | 47 | 14 | 28 |
| Q03 | Passing urine frequently | 29 | 67 | 28 | 56 |
| Q04 | Stop eating | 20 | 47 | 25 | 50 |
| Q05 | Dry mouth | 20 | 47 | 23 | 46 |
| **Q06** | **Memory issues** | **28** | 65 | 31 | 62 |
| Q07 | Fits | 3 | 7 | 2 | 4 |
| Q08 | Wet the bed | 3 | 7 | 10 | 20 |
| Q09 | **Feel tired** | **33** | 77 | 41 | 82 |
| **Q10** | Erection problems | 15 | 35 | 6 | 12 |
| Q11 | Thirsty | 29 | 67 | 28 | 56 |
| Q12 | Bloated body | 16 | 37 | 22 | 44 |
| Q13 | Problems passing water | 6 | 14 | 6 | 12 |
| Q14 | Palpitations | 23 | 54 | 20 | 40 |
| Q15 | **Shaky hands** | **19** | 44 | 15 | 30 |
| **Q16** | Lost sex drive | 23 | 54 | 13 | 26 |
| Q17 | **Tremors** | **8** | 19 | 10 | 20 |
| **Q18** | Fluid from nipples | 2 | 5 | 3 | 6 |
| Q19 | **Drooling mouth** | **19** | 44 | 31 | 62 |
| **Q20** | **Faint when stand up** | **18** | 42 | 20 | 40 |
| **Q21** | Blurry vision | 22 | 51 | 22 | 44 |
| Q22 | **Restlessness** | **25** | 58 | 26 | 52 |
| **Q23** | Put weight on | 30 | 70 | 31 | 62 |
| Q24 | Slower | 24 | 56 | 27 | 54 |
| Q25 | Oversleep | 26 | 61 | 28 | 56 |
| Q26 | Reduced ejaculation | 13 | 30 | 5 | 10 |
| Q27 | Joints hurt | 20 | 47 | 19 | 38 |
| Q28 | Can't stay awake | 22 | 51 | 25 | 50 |
| Q29 | Diabetes | 3 | 7 | 3 | 6 |
| Q30 | **Muscle spasms/cramps** | **19** | 44 | 17 | 34 |
| **Q31** | Vertigo | 9 | 21 | 13 | 26 |
| Q32 | Breasts | 8 | 19 | 2 | 4 |
| Q33 | Rash | 7 | 16 | 10 | 20 |
| Q34 | Feverish | 9 | 21 | 9 | 18 |
| Q35 | **Not enjoying sex** | **17** | 40 | 9 | 18 |
| **Q36** | Slowed down thought | 26 | 61 | 22 | 44 |
| Q37 | **Period** | **9** | 21 | 8 | 16 |
| **Q38** | Constipated | 13 | 30 | 20 | 40 |
| Q39 | **Stiff muscles** | **14** | 33 | 16 | 32 |
| **Q40** | Feel drugged | 17 | 40 | 16 | 32 |
| Q41 | **Restless legs** | **11** | 26 | 20 | 40 |
| **Q42** | Feel sick | 17 | 40 | 16 | 32 |
| Q43 | **Hard to fall asleep** | **21** | 49 | 18 | 36 |
| **Q44** | Catatonia | 4 | 9 | 4 | 8 |
| Q45 | **Sweating** | **17** | 40 | 17 | 34 |
| **Q46** | **Coordination issues** | **16** | 37 | 12 | 24 |
| **Q47** | **Hard to concentrate** | **26** | 61 | 31 | 62 |
| **Q48** | Stuffy nose | 20 | 47 | 21 | 42 |
| Q49 | Diarrhoea | 12 | 28 | 12 | 24 |
| Q50 | **Depressed** | **24** | 56 | 27 | 54 |
| **Q51** | Jaw locks up | 9 | 21 | 6 | 12 |
| Q52 | Sensitivity to sunlight | 9 | 21 | 13 | 26 |
| Q53 | Hard to get out of bed | 26 | 61 | 30 | 60 |
| Q54 | Pain in kidney | 12 | 28 | - | - |

Web Table 3: Descriptive indices of the four sub scores –Sample 2.

|  | ***Time 1*** | | | ***Time 2*** | | | ***Comparison (Wilcoxon test)*** | |
| --- | --- | --- | --- | --- | --- | --- | --- | --- |
|  | ***Mean (sd)*** | ***Median*** | ***range*** | ***Mean (sd)*** | ***Median*** | ***range*** | ***Z*** | ***p-value*** |
| **Total side effects** | 21.0 (11.5) | 19.0 | 0 - 47 | 21.4 (13) | 20.0 | 0 - 52 | -0.238 | 0.812 |
| **Intensity** | 36.3 (24.1) | 31.0 | 0 - 95 | 34.8 (25.3) | 29.0 | 0 - 108 | -1.050 | 0.294 |
| **Life impact** | 32.3 (26.5) | 24.0 | 0 - 94 | 32.5 (27.6) | 22.0 | 2 - 109 | -0.009 | 0.993 |
| **Distress** | 6.0 (8.2) | 2.0 | 0 - 30 | 6.2 (8.3) | 1.0 | 0 - 29 | -0.474 | 0.636 |

Web Table 4: Spearman’s correlation coefficients between the three subscale scores and the total number of side effects (Time 1 below and Time 2 above the main diagonal).

|  | **Total side effects** | | | **Intensity** | | | **Life impact** | | | **Distress** | | |
| --- | --- | --- | --- | --- | --- | --- | --- | --- | --- | --- | --- | --- |
|  | rho | p | N | rho | p | N | rho | p | N | rho | p | N |
| **Total side effects** |  |  |  | 0.93 | <0.001 | 43 | 0.87 | <0.001 | 37 | 0.38 | 0.015 | 41 |
| **Intensity** | 0.92 | <0.001 | 43 |  |  |  | 0.87 | <0.001 | 37 | 0.48 | 0.002 | 41 |
| **Life impact** | 0.89 | <0.001 | 37 | 0.91 | <0.001 | 37 |  |  |  | 0.51 | <0.001 | 37 |
| **Distress** | 0.42 | 0.006 | 41 | 0.50 | 0.001 | 41 | 0.48 | 0.002 | 37 |  |  |  |

Web Table 5: Correlation coefficients between the total scores and age along with gender comparisons.

|  | ***Sample 2*** | | | | ***Sample 3*** | |
| --- | --- | --- | --- | --- | --- | --- |
|  | ***Time 1*** | | ***Time 2*** | |  |  |
|  | ***age*** | ***gender**** | ***age*** | ***gender**** | ***age*** | ***gender**** |
|  | ***r***  ***(p-value)*** | ***MD (s.e.),***  ***p-value*** | ***r***  ***(p-value)*** | ***MD (s.e.),***  ***p-value*** | ***r***  ***(p-value)*** | ***MD (s.e.),***  ***p-value*** |
| **Total side effects** | -0.1  (0.358) | -0.2 (3.7), 0.962 | -0.2  (0.223) | 2.6 (4.1),  0.532 | 0  (0.799) | -1.7 (2.7),  0.273 |
| **Intensity** | -0.1  (0.442) | -3.8 (7.7),  0.625 | -0.1  (0.484) | 0.9 (8.1), 0.908 | 0.1  (0.468) | -3.8 (6.1),  0.323 |
| **Life impact** | -0.1  (0.616) | -0.3 (9),  0.769 | -0.1  (0.573) | -1.6 (9.5),  0.888 | 0.3  (0.076) | -6.1 (5.9),  0.282 |
| **Distress** | 0.1  (0.432) | -0.1 (2.7),  0.677 | 0.1  (0.526) | 1 (2.7),  0.760 | 0.2  (0.121) | --1.7 (1.9),  0.202 |
| *Mann-Whitney test | | | | | | |

Web Table 6: Cohen’s Kappa coefficient κ (for the binary items) and weighted Cohen’s Kappa coefficient κw (for the ordinal items), between the two time points (confidence intervals within brackets).

|  | | **Intensity** | | **Distress** | | **Life impact** | |
| --- | --- | --- | --- | --- | --- | --- | --- |
| **Item** | **Side effect** | **N** | **κ_w_ (CI)** | **N** | **κ (CI)** | **N** | **κ_w_ (CI)** |
| Q01 | Headaches | 43 | 0.7 (0.5, 0.9) | 17 | 0.9 (0.6, 1.0) | 17 | 0.5 (0.2, 0.9) |
| Q02 | Not feeling emotions | 43 | 0.6 (0.4, 0.8) | 15 | 0.8 (0.5, 1.0) | 15 | 0.4 (0.2, 0.7) |
| Q03 | Passing urine frequently | 42 | 0.7 (0.5, 0.9) | 28 | *0.3 (-0.1, 0.7) | 26 | 0.6 (0.4, 0.8) |
| Q04 | Stop eating | 43 | 0.6 (0.3, 0.8) | 18 | 0.6 (0.1, 1.0) | 18 | 0.7 (0.5, 1.0) |
| Q05 | Dry mouth | 43 | 0.6 (0.3, 0.8) | 19 | 0.6 (0.0, 1.0) | 17 | 0.5 (0.1, 0.9) |
| Q06 | Memory issues | 43 | 0.8 (0.7, 0.9) | 28 | 0.9 (0.7, 1.0) | 25 | 0.5 (0.2, 0.8) |
| Q07 | Fits | 43 | * 0.5 (-0.1, 1.1) | 2 | (b) | 2 | (b) |
| Q08 | Wet the bed | 42 | 0.9 (0.7, 1.1) | 2 | (b) | 2 | (b) |
| Q09 | Feel tired | 43 | 0.8 (0.7, 0.9) | 29 | 0.7 (0.4, 1.0) | 26 | 0.7 (0.4, 0.9) |
| Q10 | Erection problems | 28 | 0.8 (0.6, 1.0) | 14 | 0.5 (0.1, 0.8) | 12 | 0.8 (0.5, 1.0) |
| Q11 | Thirsty | 43 | 0.6 (0.4, 0.8) | 25 | *0.3 (-0.2, 0.9) | 23 | 0.6 (0.3, 0.9) |
| Q12 | Bloated body | 43 | 0.8 (0.7, 1.0) | 14 | 0.6 (0.1, 1.0) | 13 | 0.5 (0.1, 0.9) |
| Q13 | Problems passing water | 42 | 0.4 (0.1, 0.8) | 5 | 1 | 5 | (b) |
| Q14 | Palpitations | 43 | 0.7 (0.5, 0.9) | 18 | 0.7 (0.3, 1.0) | 16 | 0.5 (0.1, 0.9) |
| Q15 | Shaky hands | 43 | 0.8 (0.7, 0.9) | 16 | *0.2 (-0.4, 0.7) | 14 | *0.1 (-0.2, 0.5) |
| Q16 | Lost sex drive | 43 | 0.6 (0.4, 0.9) | 18 | 0.6 (0.3, 1.0) | 16 | 0.8 (0.6, 1.0) |
| Q17 | Tremors | 43 | 0.6 (0.2, 1.0) | 7 | (a) | 7 | (a) |
| Q18 | Fluid from nipples | 43 | 0.8 (0.5, 1.1) | 2 | (b) | 2 | (b) |
| Q19 | Drooling mouth | 43 | 0.9 (0.9, 1.0) | 17 | 0.8 (0.3, 1.0) | 15 | 0.7 (0.3, 1.1) |
| Q20 | Faint when stand up | 43 | 0.7 (0.4, 0.9) | 13 | *0.3 (-0.2, 0.9) | 11 | 1.0 (0.9, 1.1) |
| Q21 | Blurry vision | 43 | 0.7 (0.5, 0.9) | 17 | 0.5 (0.1, 0.9) | 16 | 0.5 (0.0, 0.9) |
| Q22 | Restlessness | 43 | 0.7 (0.5, 0.9) | 18 | 0.5 (0.1, 0.9) | 18 | 0.9 (0.8, 1.0) |
| Q23 | Put weight on | 43 | 0.5 (0.2, 0.8) | 25 | 0.9 (0.8, 1.0) | 22 | 0.9 (0.8, 1.0) |
| Q24 | Slower | 43 | 0.5 (0.2, 0.8) | 20 | 0.6 (0.1, 1.0) | 18 | 0.5 (0.2, 0.8) |
| Q25 | Oversleep | 43 | 0.6 (0.4, 0.9) | 21 | 0.5 (0.1, 0.8) | 19 | 0.5 (0.1, 0.9) |
| Q26 | Reduced ejaculation | 29 | 0.6 (0.4, 0.9) | 9 | 1 | 8 | 0.9 (0.7, 1.1) |
| Q27 | Joints hurt | 43 | 0.8 (0.6, 0.9) | 15 | 0.5 (0.1, 0.9) | 14 | 0.7 (0.5, 1.0) |
| Q28 | Can't stay awake | 43 | 0.7 (0.5, 0.9) | 18 | 0.5 (0.1, 0.9) | 16 | 0.7 (0.3, 1.0) |
| Q29 | Diabetes | 43 | 0.9 (0.8, 1.0) | 3 | (b) | 3 | (b) |
| Q30 | Muscle spasms/cramps | 43 | 0.4 (0.1, 0.7) | 12 | 0.7 (0.3, 1.0) | 9 | 0.8 (0.5, 1.1) |
| Q31 | Vertigo | 43 | 0.8 (0.5, 1.0) | 6 | 1 | 6 | *0.3 (-0.4, 1.0) |
| Q32 | Breasts | 32 | 0.9 (0.7, 1.0) | 7 | 0.7 (0.2, 1.0) | 7 | 0.6 (0.1, 1.0) |
| Q33 | Rash | 43 | *0.3 (-0.1, 0.7) | 3 | (b) | 3 | (b) |
| Q34 | Feverish | 43 | 0.8 (0.6, 1.0) | 7 | *0.6 (-0.1, 1.0) | 7 | 0.8 (0.5, 1.1) |
| Q35 | Not enjoying sex | 37 | 0.7 (0.4, 0.9) | 14 | 0.5 (0.1, 1.0) | 12 | 0.9 (0.7, 1.0) |
| Q36 | Slowed down thought | 43 | 0.6 (0.4, 0.9) | 20 | 0.8 (0.5, 1.0) | 17 | 0.7 (0.5, 0.9) |
| Q37 | Period | 11 | 0.5 (0.0, 1.0) | 8 | (a) | 6 | *0.3 (-0.1, 0.8) |
| Q38 | Constipated | 43 | 0.9 (0.8, 1.0) | 12 | 0.7 (0.3, 1.0) | 12 | 0.8 (0.7, 1.0) |
| Q39 | Stiff muscles | 43 | 0.8 (0.6, 1.0) | 12 | 0.5 (0.0, 1.0) | 12 | 0.8 (0.6, 0.9) |
| Q40 | Feel drugged | 43 | 0.8 (0.6, 1.0) | 15 | *0.3 (-0.2, 0.8) | 14 | 0.6 (0.2, 1.0) |
| Q41 | Restless legs | 43 | 0.9 (0.8, 1.0) | 10 | *0.3 (-0.2, 0.8) | 9 | 0.8 (0.7, 1.0) |
| Q42 | Feel sick | 43 | 0.6 (0.3, 0.9) | 11 | 0.6 (0.1, 1.0) | 9 | 0.9 (0.7, 1.0) |
| Q43 | Hard to fall asleep | 43 | 0.6 (0.4, 0.9) | 19 | 0.8 (0.5, 1.0) | 17 | 0.6 (0.2, 1.0) |
| Q44 | Catatonia | 43 | *0.3 (-0.1, 0.7) | 2 | (b) | 2 | 1 (1, 1.0) |
| Q45 | Sweating | 43 | 0.6 (0.3, 0.9) | 12 | 1 | 10 | 0.6 (0.1, 1.1) |
| Q46 | Coordination issues | 43 | 0.8 (0.6, 1.0) | 13 | 0.7 (0.3, 1.0) | 12 | 0.6 (0.1, 1.1) |
| Q47 | Hard to concentrate | 43 | 0.7 (0.5, 0.9) | 23 | 1 | 20 | 0.8 (0.7, 0.9) |
| Q48 | Stuffy nose | 43 | 0.7 (0.5, 0.9) | 17 | *0.4 (-0.1, 0.8) | 16 | 0.8 (0.6, 1.0) |
| Q49 | Diarrhoea | 43 | 0.4 (0.0, 0.8) | 9 | *0.6 (-0.1, 1.0) | 7 | 0.6 (0.2, 0.9) |
| Q50 | Depressed | 43 | 0.7 (0.5, 0.9) | 23 | 0.5 (0.1, 0.9) | 21 | 0.7 (0.5, 0.9) |
| Q51 | Jaw locks up | 43 | 0.7 (0.4, 0.9) | 7 | *0.3 (-0.5, 1.0) | 6 | *0.2 (-0.6, 1.1) |
| Q52 | Sensitivity to sunlight | 43 | 0.7 (0.6, 0.9) | 8 | *-0.2 (-0.5, 1.0) | 8 | *0.2 (-0.3, 0.8) |
| Q53 | Hard to get out of bed | 43 | 0.7 (0.6, 0.8) | 22 | 0.5 (0.2, 0.9) | 20 | 0.8 (0.6, 1.0) |
| (a) Kappa cannot be computed since at least one of the two variables is constant.  (b) Not enough data to compute the coefficient.  * Non-significant. | | | | | | | |

Web Table 7: Descriptive indices of the four sub scores –Sample 3.

|  | ***N*** | ***Mean*** | ***Median*** | ***SD*** | ***range*** |
| --- | --- | --- | --- | --- | --- |
| Total side effects | 50 | 18.5 | 19.5 | 9.4 | 2-43 |
| Intensity | 50 | 34.0 | 33.0 | 21.3 | 2-92 |
| Life impact | 50 | 28.2 | 28.5 | 20.9 | 0-95 |
| Distress | 50 | 5.5 | 3.0 | 6.6 | 0-32 |

**Web Table 8 Items mentioned among the three most important ones, per sample and in total.**

| **Side effect** | **Sample 2**  **(N=43)** | | **Sample 3**  **(N=50)** | | **Total**  **(N=93)** | |
| --- | --- | --- | --- | --- | --- | --- |
|  | **N** | ***%*** | **N** | **%** | **N** | **%** |
| 01) Headaches | 4 | *9.3* | 2 | *4* | 6 | *6.5* |
| 02) Not feeling emotions | 1 | *2.3* | 0 | *0* | 1 | *1.1* |
| 03) Passing urine frequently | 3 | *7* | 4 | *8* | 7 | *7.5* |
| 04) Stop eating | 2 | *4.7* | 2 | *4* | 4 | *4.3* |
| 05) Dry mouth | 2 | *4.7* | 2 | *4* | 4 | *4.3* |
| 06) Memory issues | 7 | *16.3* | 2 | *4* | 9 | *9.7* |
| 07) Fits | 0 | *0* | 0 | *0* | 0 | *0* |
| 08) Wet the bed | 1 | *2.3* | 0 | *0* | 1 | *1.1* |
| 09) Feel tired | **10** | ***23.3*** | **9** | ***18*** | **19** | ***20.4*** |
| 10) Erection problems | 4 | *9.3* | 0 | *0* | 4 | *4.3* |
| 11) Thirsty | 2 | *4.7* | 0 | *0* | 2 | *2.2* |
| 12) Bloated body | 0 | *0* | 0 | *0* | 0 | *0* |
| 13) Problems passing water | 0 | *0* | 0 | *0* | 0 | *0* |
| 14) Palpitations | 1 | *2.3* | 3 | *6* | 4 | *4.3* |
| 15) Shaky hands | 1 | *2.3* | 3 | *6* | 4 | *4.3* |
| 16) Lost sex drive | 3 | *7* | 1 | *2* | 4 | *4.3* |
| 17) Tremors | 1 | *2.3* | 1 | *2* | 2 | *2.2* |
| 18) Fluid from nipples | 0 | *0* | 0 | *0* | 0 | *0* |
| 19) Drooling mouth* | **5** | ***11.6*** | **16** | ***32*** | **21** | ***22.6*** |
| 20) Faint when stand up | 1 | *2.3* | 1 | *2* | 2 | *2.2* |
| 21) Blurry vision | 4 | *9.3* | 4 | *8* | 8 | *8.6* |
| 22) Restlessness | 3 | *7* | 2 | *4* | 5 | *5.4* |
| 23) Put weight on | **6** | ***14*** | **7** | ***14*** | **13** | ***14*** |
| 24) Slower | 0 | *0* | 1 | *2* | 1 | *1.1* |
| 25) Oversleep | 3 | *7* | 4 | *8* | 7 | *7.5* |
| 26) Reduced ejaculation | 0 | *0* | 0 | *0* | 0 | *0* |
| 27) Joints hurt | 4 | *9.3* | 4 | *8* | 8 | *8.6* |
| 28) Can't stay awake | 4 | *9.3* | 3 | *6* | 7 | *7.5* |
| 29) Diabetes | 0 | *0* | 1 | *2* | 1 | *1.1* |
| 30) Muscle spasms/cramps | 0 | *0* | 2 | *4* | 2 | *2.2* |
| 31) Vertigo | 4 | *9.3* | 5 | *10* | 9 | *9.7* |
| 32) Breasts | 0 | *0* | 0 | *0* | 0 | *0* |
| 33) Rash | 0 | *0* | 0 | *0* | 0 | *0* |
| 34) Feverish | 0 | *0* | 1 | *2* | 1 | *1.1* |
| 35) Not enjoying sex | 0 | *0* | 1 | *2* | 1 | *1.1* |
| 36) Slowed down thought | 3 | *7* | 1 | *2* | 4 | *4.3* |
| 37) Period | 0 | *0* | 0 | *0* | 0 | *0* |
| 38) Constipated | 2 | *4.7* | 5 | *10* | 7 | *7.5* |
| 39) Stiff muscles | 1 | *2.3* | 0 | *0* | 1 | *1.1* |
| 40) Feel drugged | 1 | *2.3* | 0 | *0* | 1 | *1.1* |
| 41) Restless legs | 0 | *0* | 1 | *2* | 1 | *1.1* |
| 42) Feel sick | 2 | *4.7* | 0 | *0* | 2 | *2.2* |
| 43) Hard to fall asleep | 3 | *7* | 5 | *10* | 8 | *8.6* |
| 44) Catatonia | 0 | *0* | 0 | *0* | 0 | *0* |
| 45) Sweating | 3 | *7* | 4 | *8* | 7 | *7.5* |
| 46) Coordination issues | 0 | *0* | 0 | *0* | 0 | *0* |
| 47) Hard to concentrate | 2 | *4.7* | 4 | *8* | 6 | *6.5* |
| 48) Stuffy nose | 0 | *0* | 0 | *0* | 0 | *0* |
| 49) Diarrhoea | 0 | *0* | 1 | *2* | 1 | *1.1* |
| 50) Depressed | 3 | *7* | 1 | *2* | 4 | *4.3* |
| 51) Jaw locks up | 0 | *0* | 0 | *0* | 0 | *0* |
| 52) Sensitivity to sunlight | 0 | *0* | 0 | *0* | 0 | *0* |
| 53) Hard to get out of bed | 3 | *7* | 0 | *0* | 3 | *3.2* |
| **Significant difference: chi-square=5.488, df=1, p=0.019* | | | | | | |
